# Coffee and Tobacco

**Published:** 1857-12

**Authors:** 


### WHERE ARE THEY?

THE literary men of France, who were young a quarter of a century ago? The Paris correspondent of the *Boston Traveller* writes:

"*De Balzac* is dead! Coffee killed him.

"*Frederic Soulie* is dead; the victim of coffee and licentiousness.

"*Eugene Briffaut* died a madman in the Chazenton Lunatic Asylum.

"*Granville* became insane, and breathed his last in a private madhouse.

"*Lassally* died a raving lunatic.

"*Lowe Weimars* died from licentiousness and opium-eating.

"*Charles de Bernard* died from coffee and licentiousness.

"*Henri Boyle* died from coffee and women.

"*Hippolite Royal Collard* died from tobacco and coffee.

"*Gerard de Nerval*, after oscillating between plenty and want, abstemiousness and licentiousness, went mad, and hung himself.

"*Rabbe*, after suffering a thousand deaths from a loathsome disease, took poison to end his prolonged torture.

"*Alfred de Musset* died a victim to the bottle and cigar.

"*Count Alfred D'Orsay* was killed by the cigar and licentiousness.

"*Eugene Sue:* coffee and women were his ruin.

"All mowed down in the prime of life, in the meridian of their intellect and fame!"

*La Belle Paris!* the synonym of all that is beautiful; the city of gayety and revelry, of music and of mirth, where pleasure lures, dazzles, intoxicates, and then destroys! is this the sad end to which your young men of culture and intellect arrive, in a short quarter of a century? Then let it be a loud warning to the youth of our own time and nation, that a better path is marked out for them in that *Book of all books*, which counsels to be *temperate in all things*, to take hold of *wisdom*, whose ways are ways of pleasantness—whose paths are peace.

